# Cementless total hip arthroplasty in patients with ankylosing spondylitis: A retrospective observational study

**DOI:** 10.1097/MD.0000000000005813

**Published:** 2017-01-27

**Authors:** Jun Xu, Min Zeng, Jie Xie, Ting Wen, Yihe Hu

**Affiliations:** a Department of Orthopedics, Seventh People's Hospital of Shenzhen, Shenzhen, Guangdong; b Department of Orthopedics, Xiangya Hospital, Central South University, Changsha, Hunan, China.

**Keywords:** ankylosing spondylitis, arthroplasty, hip, surgery

## Abstract

Controversies on the surgical protocols and efficacies of total hip arthroplasty (THA) in ankylosing spondylitis (AS) still exist. The aim of this study was to retrospectively analyze the perioperative managements and their outcomes related to performing THA on patients with AS.

Data of 54 AS patients who underwent 81 THAs between 2008 and 2014 were retrospectively analyzed. Clinical and imaging data were collected preoperatively, postoperatively, and during the follow-up period for surgical efficacy.

Using posterolateral approach, cementless prostheses were selected in all cases. Mean follow-up period was 3.6 years (range, 2–8 years). Inclinations and anteversions of acetabular cups were 36.3°±4.5° (range, 30°–50°) and 12.3°±4.9° (range, 0°–25°) respectively. Mean visual analog scale (VAS) score decreased from 6.7 ± 2.1 (range, 4–10) preoperatively to 1.5 ± 1.0 (range, 0–4) at final follow-up, and mean Harris hip score (HHS) improved from 31.2 ± 11.6 (range, 15–45) to 86.1 ± 4.3 (range, 80–95) (*P* < 0.05). Postoperative range of motion (ROM) in flexion was improved from 6.7°±13.5° (range, 0°–50°) preoperatively to 82.5°±6.4° (range, 70°–100°) at final follow-up, and ROM in extension was improved from 1.8°±5.7°(range, 0°–15°) to 15.4°±2.6° (range, 10°–20°) (*P* < 0.05). Heterotopic ossification (HO) was documented in 9 hips (11.1%). Signs of stable fibrous ingrowth and bone ingrowth were detected in 52 and 29 hips, respectively. Sciatic never injury was occurred in 3 cases, and treated conservatively. There were no signs of periprosthetic fractures, dislocation, or prosthesis loosening.

Surgical efficacies of THA for AS patients with severe hip involvement are satisfactory.

## 1 Introduction

Ankylosing spondylitis (AS) is a chronic inflammatory disease with an unclear etiology. While AS primarily involves the spine and sacroiliac joints of patients 20 to 30 years of age, the appendicular skeleton is frequently involved.^[1]^ The global prevalence of AS is between 0.1% and 1.4%.^[2]^ The hip is the most frequently involved large peripheral joint, with pain, swelling, and deformity in 25% to 50% of AS patients.^[3]^ A severe hip deformity can seriously impact the quality of life of affected patients, and the most well-accepted treatment of choice is total hip arthroplasty (THA).^[4,5]^ However, the optimal THA techniques in the setting of AS and subsequent surgical outcomes have been poorly characterized.

The objective of this study was to perform a retrospective study evaluating the surgical techniques and outcomes of THA for AS patients. Factors of particular interest included surgical timing, prosthesis selection, intraoperative managing strategies, heterotopic ossification (HO) prophylaxis and subsequent occurrence, clinical and radiographic evaluation, complication, and its managements.

## 2 Material and methods

### 2.1 Patients and clinical data

A retrospective analysis of 54 AS patients with severe hips involvement in 81 hips treated by THAs was conducted between January 2008 and September 2014 in Xiangya Hospital Central South University. With the help of a rheumatologist, the clinical diagnosis of AS was made using 1984 modified New York criteria.^[6]^ The main indications for THA in these AS patients included intractable pain that failed to conservative treatment, and loss of motion and poor posture that is unable to function independently. For patients with elevated levels of inflammatory biomarkers before operation, infection was ruled out by clinical manifestations and necessary auxiliary examinations. For patients with systematic involvements, with the help of anesthesiologist, cardiologist, rheumatologist, pulmonary and rehabilitation physician, the clinical condition and surgical risks of AS patients were preoperative evaluated and properly adjusted. For patients with severe spinal deformity, a spinal osteotomy was performed prior to THA. Three-dimensional reconstruction computed tomography scans of affected hip were used for better anatomical reconstruction intraoperatively.

### 2.2 Surgical data

Under epidural anesthesia, all THAs were performed using posterolateral approach by a single senior surgeon. Simultaneous bilateral THAs were completed for patients with bilateral involvements, and the hip with more severe pathology was replaced first. For patients without hip ankylosis, femoral neck osteotomy was carried out after hip subluxation or complete luxation gently, while for the rest of surgeries, a 2-step in situ osteotomy technique was performed after clearly identifying the boundary between the femoral head and acetabulum. No trochanteric osteotomy was preformed. By using the foveal soft tissue as landmark for locating the original joint plane, the true acetabulm was exposed after removal of residual femoral head and surrounding soft tissue. Cementless prosthesis was used in all cases. Intraoperative radiographs and repeated trial reductions were performed to minimize the rate of prosthetic malpositioning. The transverse acetabular ligament and lesser trochanter were used for component orientation. A decreased anteversion of the acetabular cup and an increased anteversion of the femoral component were indicated for internal rotation, and an increased anteversion of the acetabular cup and a decreased anteversion of the femoral component for external rotation deformities. And a reduced inclination of the acetabular cup was indicated for adduction deformity. All layers of the incision were sutured after placing of drainage. Intraoperative autologous blood transfusion and homologous blood was prepared to prevent hemorrhagic complication.

After surgeries, a prophylactic regimen of cefazolin was administered for 48 hours and rivaroxaban for 2 weeks for prophylaxis of deep vein thrombosis. No HO prophylaxis was used in our institution. The time to remove the drainage was that when the fluid was not increased, usually 24 to 48 hours postoperatively. Patients were encouraged to mobilize in bed on postoperative day 1 and to walk with toe-touch weight bearing after the drain was removed and to walk with crutches on discharge. Ambulation with full weight bearing was permitted 3 months after surgery.

### 2.3 Evaluation

Clinical data, including Harris hip score (HHS) (0–100; 100 = best function), visual analog scale (VAS) (0–10; 0 = no pain), range of motion (ROM) (0 = ankylosed hip), and complication, were collected preoperatively, postoperatively, and during the follow-up period (1, 3, 6, and 12 months postoperatively and every year thereafter). Besides, the various periods of anteroposterior and lateral radiographs were observed for prosthetic status. Specifically as follows: acetabular orientation was measured by anteroposterior imaging and CT scanning during follow up;^[7]^ on the basis of the range of ectopic bone formation around the prosthesis, HO was categorized into 4 grades;^[8]^ postoperative bone ingrowth was divided into 3 types by the approved criteria;^[9]^ the development of progressive subsidence, continuous radiolucence, or pedestal formation suggested the presence of prosthesis loosening.^[9]^ The Medical Ethics Committee of Xiangya Hospital Central South University approved this study. Consent was taken from all patients for involvement in this study including consent to use data from medical records and radiographs.

### 2.4 Statistical analysis

SPSS 19.0 (IBM Corp, Armonk, NY) was used for statistical analysis. The perioperative VAS, HHS scores, and ROM measurements were compared by paired *t* test. A significant difference was defined as *P* < 0.05.

## 3 Results

There were 49 males and 5 females, 27 of whom had severe bilateral involvements. The average follow-up of these patients was 3.6 years (range, 2–8 years), with a mean age of 38.5 years (range, 18–70 years) during surgery. Hip ankylosis was identified in 63 hips, and the ROM of the rest 18 hips was 30° (range 25–50) in flexion and 8° (range 5–15) in extension.

After surgeries, overall inclinations and anteversions of acetabular cups were 36.3°±4.5° (range, 30°–50°) and 12.3°±4.9° (range, 0°–25°) respectively. Mean VAS score decreased from 6.7 ± 2.1 (range, 4–10) preoperatively to 1.5 ± 1.0 (range, 0–4) at final follow-up (*P* < 0.05). Mean HHS improved from 31.2 ± 11.6 (range, 15–45) to 86.1 ± 4.3 (range, 80–95) at final follow-up (*P* < 0.05). Postoperative ROM in flexion was improved from 6.7°±13.5° (range, 0°–50°) preoperatively to 82.5°±6.4° (range, 70°–100°) at final follow-up (*P* < 0.05), and ROM in extension was improved from 1.8°±5.7°(range, 0°–15°) to 15.4°±2.6° (range, 10°–20°) at final follow-up (*P* < 0.05).

**Figure 1:**
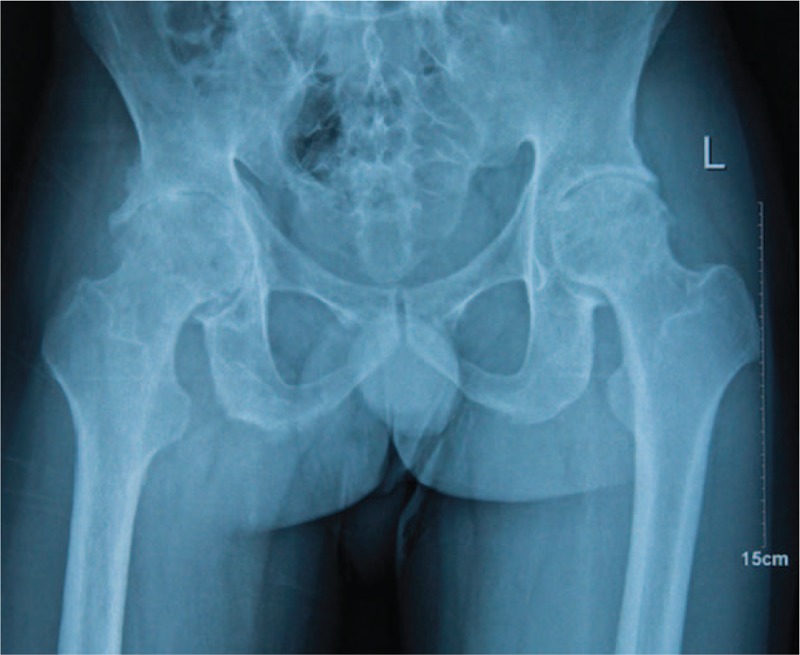
Preoperative anteroposterior radiographs of a 34-year-old man revealing narrowed joint space and marked osteophyte formation.

**Figure 2:**
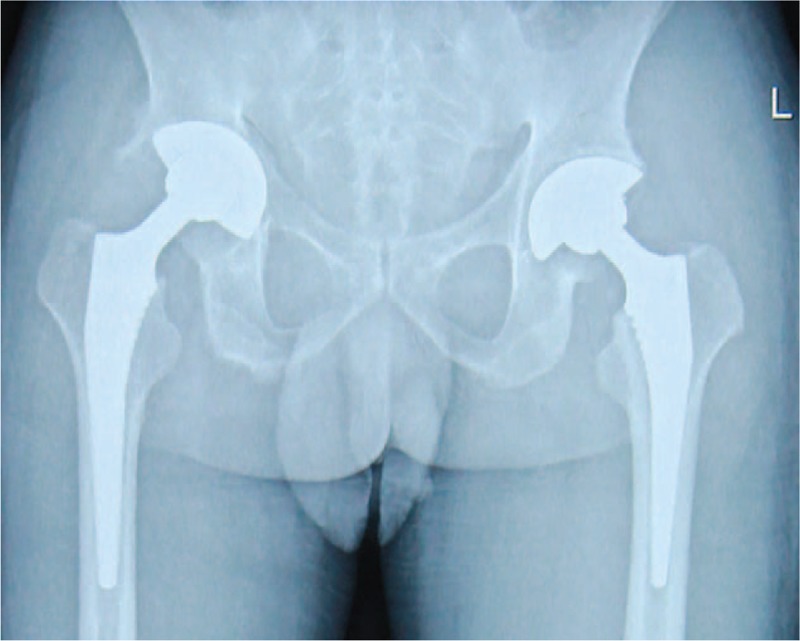
Postoperative anteroposterior radiographs of the patient 1 week after cementless THA. THA = total hip arthroplasty.

**Figure 3:**
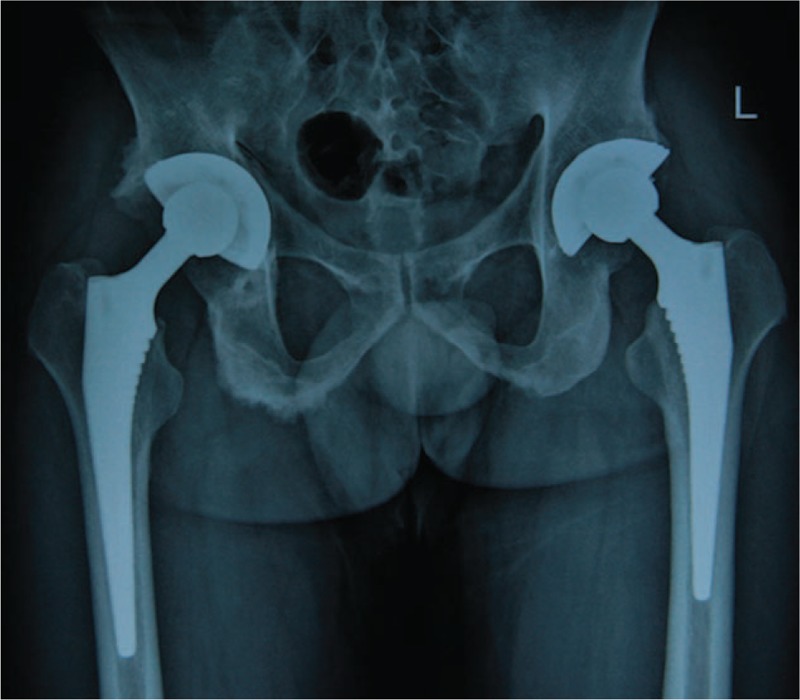
Postoperative anteroposterior radiographs of the patient 4 years after cementless THA. THA = total hip arthroplasty.

HO was documented in 9 hips (11.1%) at final follow-up, with Brooker type I ossification in 6 hips and type II in 3, and no hips were associated with a clinical complaint. There were no signs of periprosthetic fractures or dislocation during the follow-up. Sciatic never injury was occurred in 3 cases, and treated conservatively in all cases. Compared with radiographs before surgery and during follow-up, there were no signs of prosthesis loosening, and signs of stable fibrous ingrowth and bone ingrowth were detected in 52 and 29 hips, respectively, at final follow-up (Figs. 1–3).

## 4 Discussion

Despite significant advancements in the pharmacologic management of AS, THA is required for severe end-stage hip involvement of AS. The clinical results of this study showed that most patients were satisfied with the surgery having a VAS, HHS, and ROM significantly improved postoperatively. Radiographic evaluations showed favorable prosthetic location. These findings suggested that THAs can be performed on AS patients with acceptable short and mid-term benefits. However, controversies persist concerning surgical timing, implant selection, and operative technique (Table 1).^[10–21]^

**Table 1:**
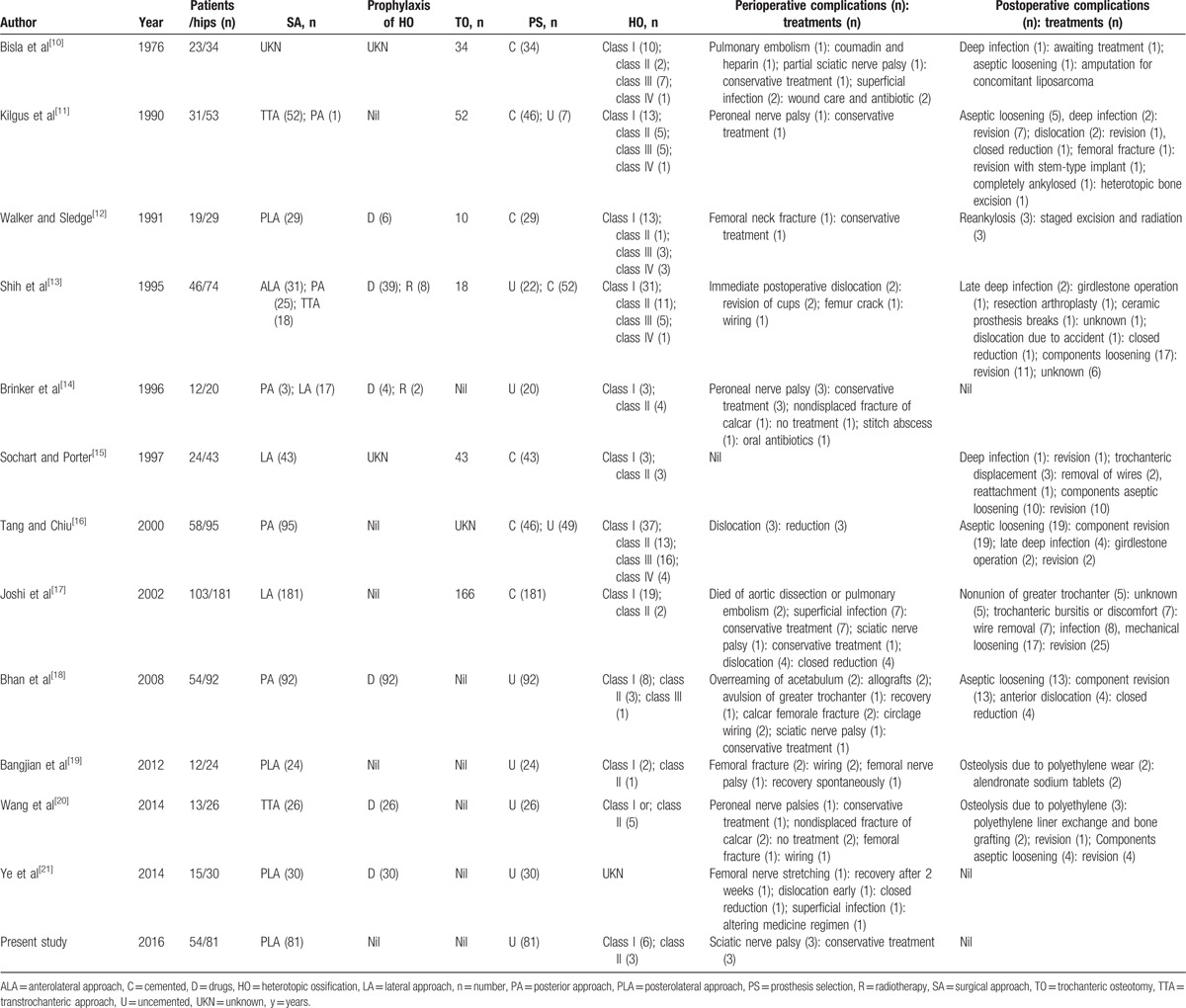
Demographic characteristics and perioperative management of the total patient population^[9–20]^.

Because of limited prosthetic durability and the generally young age of AS patients, the surgical timing of THA is a concern. The present study recommends that THA be performed on AS patients suffering from intractable pain or severe disability. The degree of functional recovery postoperatively is directly related to the patient's preoperative level of function.^[22]^ As surgical technology and implants continue to improve, age will be less of a concern for performing THA on AS patients. Patients who present with severe hip and spinal deformity are a particular challenge, and there remains no consensus on which deformity to fix first.^[10,16,19]^ Our experience has led us to agree with this assertion that a spinal osteotomy should be performed prior to THA to reduce the risk of hip dislocation.^[16,19]^ However, others suggest that a THA performed first may obviate the need for the spinal osteotomy.^[10,23]^

Significant debate has focused on the superiority of cementless or cemented components in AS patients.^[11,13,14,16,21]^ Some authors have indicated that cemented prostheses may be advantageous in AS patients, as the serious osteoporosis typically observed in affected patients makes it difficult to achieve sufficient osseointegration between bone and prosthesis.^[16,17]^ In contrast, proponents of a cementless component suggest that bone ingrowth will increase the lifespan of the implant, and reduce the difficulty of future revisions necessary in the young AS population.^[11,13,18]^ The stable ingrowth postoperatively in our research recommends cementless implants in AS patients.

As the type and degree of hip deformity differs between individual AS patients, THAs in this population are technically challenging. The choice of surgical approach, to a great extent, depended on the preference and experience of the individual surgeon, and the surgical exposure was posterolateral approach in our institution. For ankylosed hips, a 2-step in situ osteotomy technique was performed after clearly identifying the boundary between the femoral head and acetabulum. A trochanteric osteotomy was reported to improve visibility,^[10,11,13]^ although an associated increase in postoperative HO and overall operative complications (i.e., nonunion) has led to a recent move away from this technique.^[18–20]^ No trochanteric osteotomy was performed in our study.

For the varying deformities noted in AS patients, the anteversion and inclination of the implant is crucial to its initial stability and long-term survival. The consensus of previous studies is that conventional THA implant placement was associated with an increased risk of dislocation.^[21,24]^ Patients with external rotation deformity and soft tissue contracture were predisposed to anterior dislocation.^[18]^ Some theorized that pelvic hyperextension due to pelvic rotation on the sagittal plane might lead to a more anteverted and inclined acetabular cup, eventually leading to anterior dislocation.^[16,18]^ By comparing anatomic positioning with functional positioning during insertion, Tang et al^[25]^ concluded that pelvic mal-rotation on the sagittal plane caused errors in cup positioning. Thus, the standing position of the pelvis in AS patients should be noted to prevent malpositioning of the acetabular cup. We propose that a decreased anteversion of the acetabular cup and an increased anteversion of the femoral component were more appropriate for internal rotation, and an increased anteversion of the acetabular cup and a decreased anteversion of the femoral component were indicated for external rotation deformities. And a reduced inclination of the acetabular cup was indicated for adduction deformity. In addition, preoperative three-dimensional reconstruction computed tomography scans, intraoperative radiographs, and repeated trial reductions are all useful to minimize the rate of prosthetic malpositioning.

Aggressive correction of leg-length discrepancy may put the patient at risk of nerve damage. It is generally recommended that limb lengthening should be not more than 4 cm to avoid this complication.^[26]^ In our retrospective review, there were 3 cases of nerve damage, all successfully treated conservatively. AS patients may be at additional risk for nerve damage due to the increased dissection necessary to correct the soft-tissue adhesions secondary to the disease.^[14]^

HO after THA is a major challenge for AS patients. High rates of HO were reported in previous research, ranging from 11.6% to 73.7% (mean 35.2%), with a mean of 7.0% clinically important HO (Brooker classes III and IV).^[10–21]^ HO may present clinically with pain, impingement, decreased ROM, reankylosis, nerve irritation, and trochanteric bursitis.^[27]^ HO was documented in 9 hips (11.1%) in our research, with Brooker type I ossification in 6 hips and type II in 3, and no hips were associated with a clinical complaint. Prior work postulates that HO largely has to do with the pathophysiology of AS. Several of the previous studies recommended the use of nonsteroidal antiinflammatory drugs (such as indomethacin) and radiotherapy for HO prophylaxis,^[16,18,20,26]^ although others felt that the risks of prophylaxis overweighed the benefits.^[14,17]^ We assert that the aggressive prophylaxis in AS patients beyond the standard of care may not be necessary. However, when compounded with additional factors such as infection, contralateral HO, and certain surgical approaches (i.e., transtrochanteric approach), HO may remain a concern in AS patients.^[11,13]^ As the bone quality of AS patients is generally poor, caution should be exercised to avoid massive bone loss or even fracture although there were no periprosthetic fractures in our study.

The main limitations of this research are as follows: first, the length of follow-up may have been too short (range 2–8 years), although it is acceptable to study the short and med-term effects of THA in AS patients; Second, the number of cases might be too small (81 THAs), although the completeness of all clinical and radiographic data supports the findings of our study. Furthermore, our findings should be further validated using a well-powered prospective study.

## 5 Conclusion

Our studies suggest that the surgical efficacies of THA in AS patients are satisfactory. Controversies persist concerning surgical timing, implant selection, intraoperative managing strategies, and HO prophylaxis. Well-powered prospective analyses are necessary to further characterize the ideal management of this vulnerable patient population.
